# Effect of Epoxy Adhesive on Nugget Formation in Resistance Welding of SAE1004/DP600/DP780 Steel Sheets

**DOI:** 10.3390/ma11101828

**Published:** 2018-09-26

**Authors:** Yixi Zhao, Yansong Zhang, Xinmin Lai

**Affiliations:** 1 Shanghai Key Laboratory of Digital Manufacture for Thin-Walled Structures, Shanghai Jiao Tong University, Shanghai 200240, China; zhangyansong@sjtu.edu.cn; 2 State Key Laboratory of Mechanical System and Vibration, Shanghai Jiao Tong University, Shanghai 200240, China; xmlai@sjtu.edu.cn

**Keywords:** resistance welding, epoxy adhesive, finite element model, three-sheet weld-bonding, nugget size, adhesive types, bond line thickness

## Abstract

This study focused on the nugget formation in resistance welding of three dissimilar steel sheets influenced by different types and thicknesses of epoxy adhesive. An improved finite element model was employed to estimate the temperature distribution in three-sheet weld-bonding and was validated by the metallographic tests. Results showed that the weld initiation time and corresponding nugget size for weld-bonds would be earlier and larger than that of resistance spot welds in term of the same welding parameters. Compared to the adhesive Betamate Flex, the weld-bonding joint of three-sheets with adhesive Terokal 5089 would have a greater increment of the weld nugget sizes due to the increase of the static contact resistance brought by the interfaces between the steel sheets. However, the bond line thickness of the previously mentioned adhesive would take little effect on the weld sizes in weld-bonding of three dissimilar steel sheets.

## 1. Introduction

The weld-bonding process is a typical hybrid welding process that combines the technical benefits of resistance spot welding (RSW) and adhesive bonding (AB). More desirable joint performance could be produced by the joule heat and an adhesive layer acting simultaneously in one joining zone [1,2,3]. This hybrid welding process will improve the static and dynamic performance of the joint (i.e., crashworthiness, stiffness, and fatigue) at the meantime of reducing the number of resistance spot welds [4,5]. Therefore, the hybrid welding process has been widely applied in the body-in-white structures such as longitudinal rails, vertical pillars, and reinforcement beams.

With the development of epoxy adhesive production, many different types of adhesives have been made and applied in the vehicle assembly. The variety of the adhesive will lead to the different bonding conditions in terms of the various bond line thickness of the adhesive. These factors including adhesive types and bond line thickness would change the contact state between the steel sheets and result in the variation of the weld size in weld-bonding of dissimilar three-sheets. Many investigations of the weld-bonding of two steel sheets have shown that the weld size and strength of the joints could be increased due to the existence of the adhesive [6,7,8].

Further studies have proven that the increase of the weld sizes would be attributed to the increase of the contact resistance by putting the adhesive into the interfaces between the steel sheets. The adhesives will alter the current density and the joule heating generation [9,10,11,12,13]. On the contrary, the temperature field distribution caused by the generated heat then influences the contact pressure due to the thermal expansion of the steel sheets. Therefore, the nugget formation is strongly dependent on the contact states at the faying interfaces between the steel sheets. Due to the different viscosity to the steel surface, the various types and bond line thicknesses of adhesive would result in the different contact resistances at the faying interfaces. Especially in a weld-bonding joint of three-sheets, the contact states between the three sheets might become more complex than that of the two sheets, which make the joint quality difficult to control in term of the change of the adhesive types and bond line thicknesses. Therefore, it is essential that a detailed investigation of the weld-bonding of three sheets with different types and bond line thicknesses of adhesive be obtained.

In the present study, the effect of the adhesive types and bond line thickness on the nugget formation was analyzed by the analytical and simulation methods in weld-bonding of three stacks of dissimilar steel sheets. The effect of the adhesive on the contact resistance and the dynamic resistance between the steel sheets was studied by Holm contact theory and the energy conservation principle. Then, an improved finite element model was employed to investigate the temperature field distribution and the weld nugget sizes at the faying interfaces between the steel sheets in the weld-bonding process compared to the conventional resistance spot welding process. The weld initiation time and corresponding nugget size for weld-bonds will be achieved based on the model validation. Lastly, the effects of adhesive types and bond line thicknesses on the nugget sizes were assessed to determine the detailed welding process parameters and the type of the epoxy adhesive. This work would provide valuable guidelines for the selection of adhesives in the weld-bonding of three stacks of dissimilar steel sheets for body-in-white manufacturing.

## 2. Analytical Analysis

**Figure 1:**
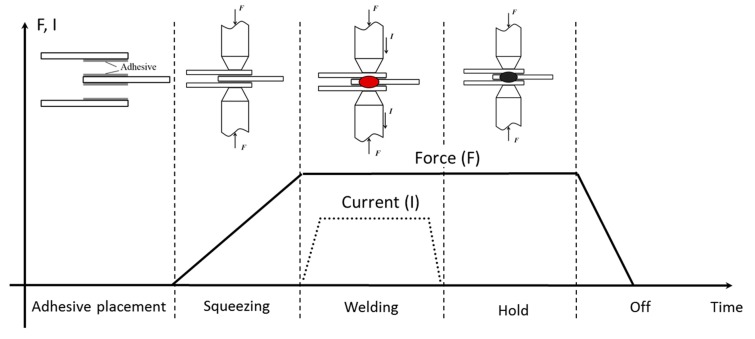
Chart for the weld-bonding process with three-sheets stacks.

Figure 1 shows a weld-bonding process composed of the following typical stages including squeeze, weld, and hold cycle. Before the squeeze cycle, the adhesive layer is first placed into the interfaces between the steel sheets. Then, the electrode force is applied to realize tight contact between the steel sheets during the squeeze cycle. In the period time, the adhesive will be squeezed out of the faying zone under the static electrode force. As the weld current is switched on to pass through the steel sheets, the weld nugget will be achieved in the weld cycle similar to the conventional RSW. Lastly, a weld-bonded joint could be formed after the cooling in the hold cycle.

### 2.1. Contact Resistance

In the squeeze cycle of the weld-bonding process, an electrode force is first applied to clamp the work pieces together. An intimate contact between the work pieces could be created to make the welding current easier to pass through. Since the adhesive would stick to the steel surface even if an electrode force is loaded, it is vital to analyze the effect of the residual adhesive on the static contact resistance at the faying area in the squeeze cycle. A local static contact resistance at the faying interface between two work pieces can be expressed by the equation below.

(1) $$Rc=\frac{\rho }{2}\sqrt{\frac{n\pi H}{F}}+\rho_{f}s_{f}\frac{nH}{F}$$

where ρ is the resistivity coefficient of the steel sheet, $\rho_{f}$ and $s_{f}$ is the film resistivity, and the film thickness, respectively. *F* is the electrode force, *n* is the total number of contact spots, and *H* is the hardness of the faying interface of the steel. In Equation (1), the first and last terms on the right-hand side represent the constriction and film resistances, respectively. In this work, the residual adhesive on the steel surface is considered as one additional film on the interface. Thus, the effect of the adhesive on the contact resistance can be expressed in a similar way to the term of the film resistances. The total contact resistance between the faying interfaces in terms of the adhesive layer could be expressed by the formula below.

(2) $$R_{CA}=\frac{\rho }{2}\sqrt{\frac{n\pi H}{F}}+\left(\rho_{f}s_{f}+k_{a}\right)\frac{nH}{F}$$

where $k_{a}$ is the influence coefficient of the adhesive, which is related to the adhesive types and the adhesive distribution on the steel surface. The existence of the residual adhesive will increase the contact resistance between the faying interfaces when compared to that without the adhesive. At the same time, this increment of the contact resistance decreases when the electrode force goes up.

### 2.2. Dynamic Resistance

**Figure 2:**
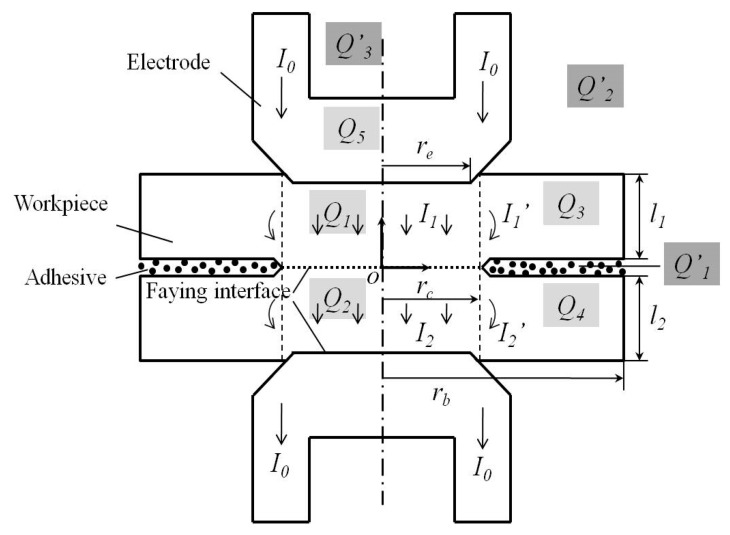
Heat generation and loss during the weld cycle in weld-bonding.

The contact resistance affected by the adhesive after the squeeze cycle would alter the joule heat generation when the welding current was applied during the weld cycle. Figure 2 shows the main heat generation (***Q*_1_**~***Q*_5_**) and loss (***Q^’^*_1_**~***Q^’^*_3_**) in a weld-bonding joint. The energy balance during the welding cycle is that the increment of the internal energy of the steel should be equal to the sum of the heat generation and loss.

To simplify the analysis, only the contacted part of the steel is considered for the internal energy and the heat generation. The other parts of the steels and the electrodes (***Q*_3_**~***Q*_5_**) are ignored for the low temperature and the small resistance during welding. Thus, the energy balance in a weld-bonding joint could be expressed by the formula below.

(3) $$cm\Delta T=I^{2}R_{D}\Delta t+Q_{l}$$

where *c* denotes the specific heat coefficient of steel, *m* is the mass of the contacted steel, Δ*T* is the temperature increment during the time Δ*t*, *I* denotes the welding current, and *R_D_* means the dynamic resistance of the weld-bonding joint including the bulk resistance of the work pieces (*R*_1_, *R*_2_) and the static contact resistance between the steel (*R_CA_*). It could be expressed by the equation shown below.

(4) $$R_{D}=R_{1}+R_{CA}+R_{2}$$

The left side term in Equation (3) means the increment of the internal energy of the welding joint. The first term on the right side of the equation denotes the joule heat generation by the dynamic resistance of the joint and the second term denotes the heat loss including the water cooling in the electrode and the air cooling around the joint. In Equation (4), it can be seen that the static resistance affected by the adhesive would increase the dynamic resistance of the weld-bonding process. Therefore, when the welding current is applied, the joule heat in weld-bonding will generate more than that without an adhesive. This result helps the temperature of the steels rise more quickly, according to Equation (3), and then the bulk resistance of the steel will increase faster with the temperature at the same time [12]. The change of the bulk resistance of the steel will influence the joule heat generation again. It can be deduced that the weld-bonding process would be greatly affected by the adhesive. The dynamic resistance might be an important and direct factor to analyze the weld-bonding process during the weld cycle. The dynamic resistance method has been used to study this weld nugget formation during the spot welding process [14,15,16,17].

## 3. Experimental Procedures

The work piece used here was a 0.8-mm-thick low carbon steel SAE1004, 1.4-mm-thick dual-phase steel DP600, and 1.8-mm-thick dual-phase steel DP780 in this study. All the steel sheets with hot-dipped galvanized (HDG) have a coating thickness of 60 g/m^2^. The chemical composition in wt % and mechanical properties of these investigated steel materials are listed in Table 1.

Two commercial available one-component epoxy resin-based adhesives (Terokal 5089, Henkel, Düsseldorf, Germany and Betamate Flex, Dow, Midland, MI, USA) were used in this study. The material properties of Terokal 5089 and Betamate Flex in the data sheet are listed in Table 2. The difference between two adhesives is that the viscosity of Betamate Flex is smaller than that of Terokal 5089.

The weld-bonding process is performed using a servo gun welding system (Obara, Yamato, Japan) with a medium frequency direct current welding machine. The electrode force with a constant value of 0 to 6.5 kN can be applied by a servo-controlled gun. A class II copper alloy with chromium and a zirconium electrode with a face diameter of 5 mm is used in the study. A weld-bonded joint was made using test coupons with approximately 100 mm long and 38 mm wide. For the single pulse weld schedule, a squeeze time of 200 ms followed by a welding time of 420 ms and a holding time of 100 ms was applied. Unless it was specifically stated, the welding current of 9.0 kA was selected to guarantee no expulsion in the weld-bonding process.

**Figure 3:**
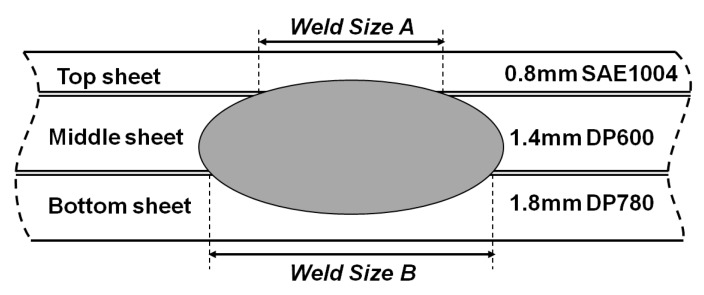
Weld nugget configuration in weld-bonding of three steel sheets.

With reference to the practical assembly example, a typical three stacks of steel sheets consisted of SAE1004 as the top sheet, DP600 as the middle sheet, and DP780 as the bottom sheet, which was shown in Figure 3. The weld diameter is often used as an evaluated indicator for spot welds. Compared to the traditional weld diameter in the center of the nugget, it is more important to determine the nugget size of the different faying interfaces for the three stack-up joint. Hence, two weld sizes A and B will be used in a weld-bonded joint quality assessment. The detailed configuration is also shown in Figure 3. These two weld sizes can be measured by an optical microscope (OM) from cross section of the weld-bonds etched with 4% Nital. The minimal acceptable weld size is 4.0 mm and 5.0 mm, respectively, for the stacks-up joint in this study, according to the American Welding Society Standard [18]. The weld sizes were prepared by the average value of five replicates for the welding tests.

## 4. Finite Element Modeling

### 4.1. Geometric Model

**Figure 4:**
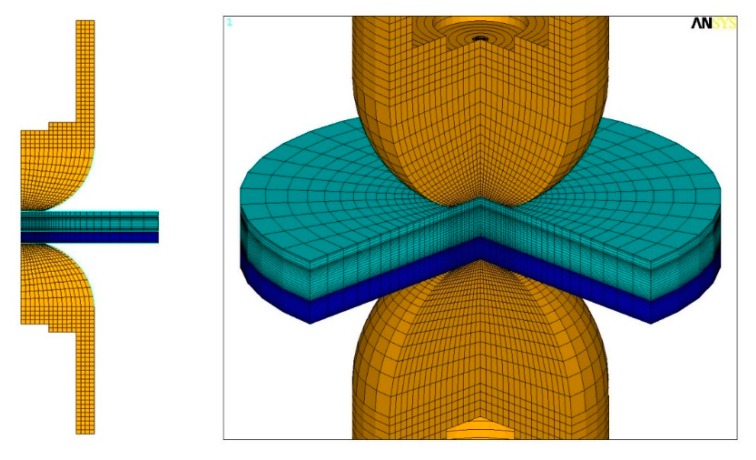
Geometrical model for the weld-bonding process.

A computational model using commercial software ANSYS 15.0 is developed to describe the coupled electrical-thermal-mechanical behavior of the weld-bonding process. The physical configuration can be simplified to a 2D axis-symmetric model. Figure 4 shows the associated element grid composed of 2656 nodes and 2651 elements in which 360 of them were contact elements, which were put into the different faying interfaces. The size of the grid graded from coarse to fine were used to capture the steep temperature gradient. Element types with PLANE67 and PLANE42 are used to analyze the electrical-thermal and thermal-mechanical behavior, respectively.

### 4.2. Boundary Conditions

**Figure 5:**
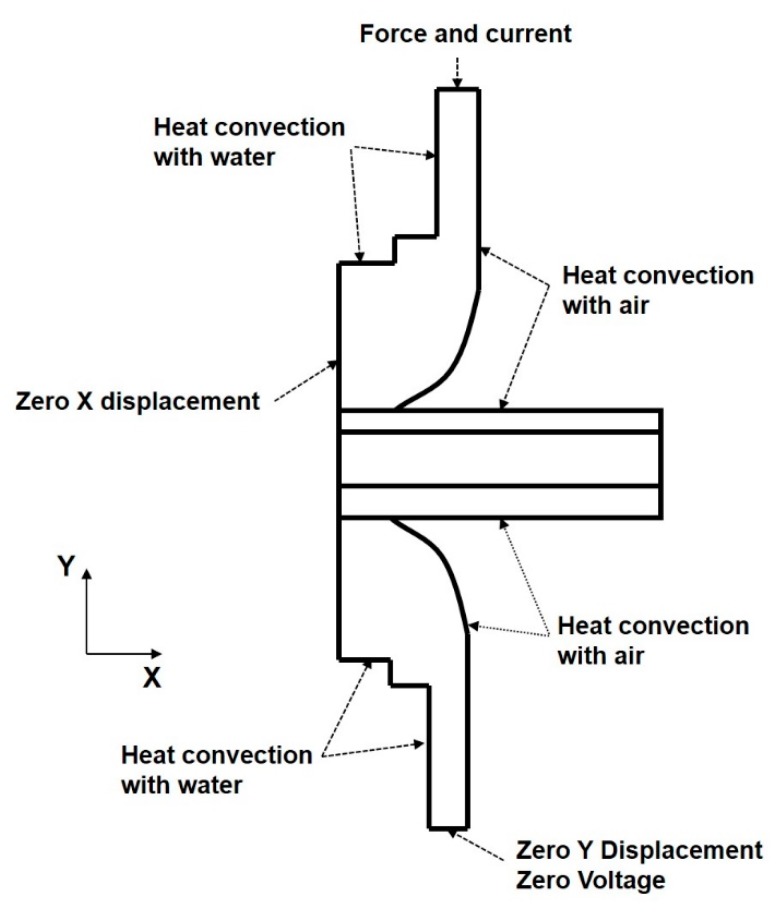
Boundary condition for modeling the weld-bonding process.

Thermal-electrical and thermal-mechanical boundary conditions, as shown in Figure 5, were applied to the finite element model. For the thermal-electrical boundary conditions, the welding current was applied uniformly to the top of the upper electrode. The electrical voltage at the bottom end of the lower electrode was set zero. The convective heat transfers to the surrounding air with a value of 19.4 W·m^−2^·K^−1^, which was used in the modeling. Both the ambient air and initial water temperature inside the copper electrode were assumed to room temperature with 21 °C. For the thermal-mechanical boundary conditions, the electrode force was applied evenly to each nodal point. The displacement of the nodes was set to zero. The symmetry line of the model was constrained to extend along the vertical axis without the lateral displacement.

### 4.3. Material Properties

In order to simulate the weld-bonding process, the relevant mechanical parameters including the modulus, the Poisson ratio, and the stress-strain relationship are needed. Similarly, some key physical (i.e., thermal conductivity, expansion coefficient, density, and specific heat) and electrical (i.e., resistivity) properties for the steel and copper are used in the coupled electrical-thermal-mechanical analysis model. The above-mentioned mechanical, electrical, and physical properties dependent on the elevated temperature assumed homogeneous and isotropic were determined from the available literature given in References [19,20].

### 4.4. Contact Model

The key parameter for calculating the weld-bonding process is the contact properties between the steels. In our work, we improved the necessary input parameter of the contact element in the model, which was defined as ***ECC*** in ***ANSYS***, by the experimental data of the dynamic resistance during the weld cycle. This is shown in the following equation.

(5) $$ECC=\frac{1}{\rho l}=\frac{1}{\left(\rho_{0}+\Delta \rho \right)\cdot l}=\frac{1}{\rho_{0}l+\left(R_{D}-R_{D}^{\prime }\right)S_{c}},$$

where $\rho_{0},l$ is the resistivity coefficient and the thickness of the contact element, respectively. $R_{D},R_{D}^{\prime }$ is the dynamic resistance of the welding process with and without adhesive, respectively. $S_{c}$ is the contact area between the steel sheets. Based on Equation (5), the influence of the adhesive on the welding process was considered in the model to analyze the nugget formation process in the weld-bonding process.

### 4.5. Computational Procedure

**Figure 6:**
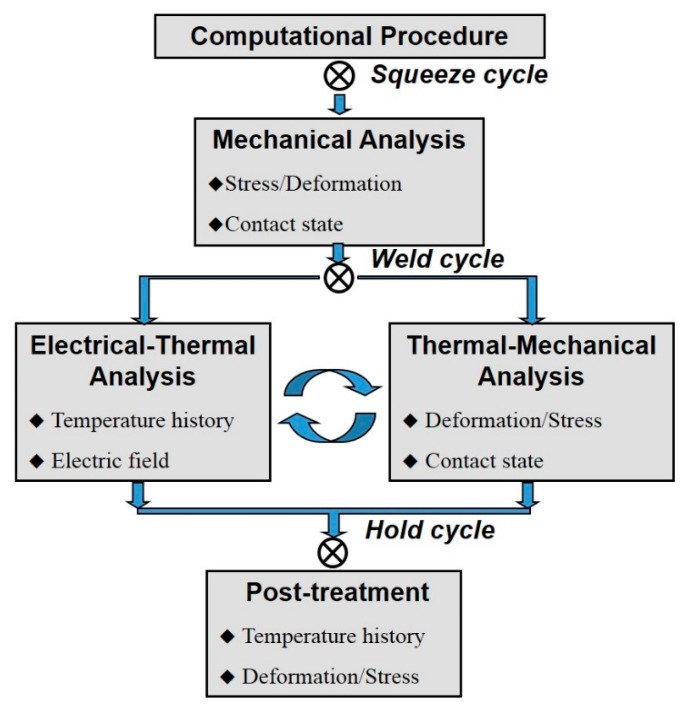
Computational procedure for finite element modeling of the weld-bonding process.

Figure 6 shows the computational procedure for modeling the weld-bonding process. Referring to Figure 6, the contact status and stress distribution from the mechanical analysis was first calculated at the squeezing stage. At the welding stage, electric-thermal-mechanical properties directly coupled to analysis is ideal for the simulation process. However, due to the computational difficulty of direct coupling, the sequentially coupled analysis in this study was adopted. The electric and temperature field obtained from the electric-thermal analysis were input into the thermal-mechanical analysis as body loading. At the same time, the contact status of the thermal-mechanical analysis was applied as the initial conditions for electric-thermal analysis. The time step of the calculated iteration is set to 20 ms.

## 5. Results and Discussion

### 5.1. Contact Resistance and Dynamic Resistance

**Figure 7:**
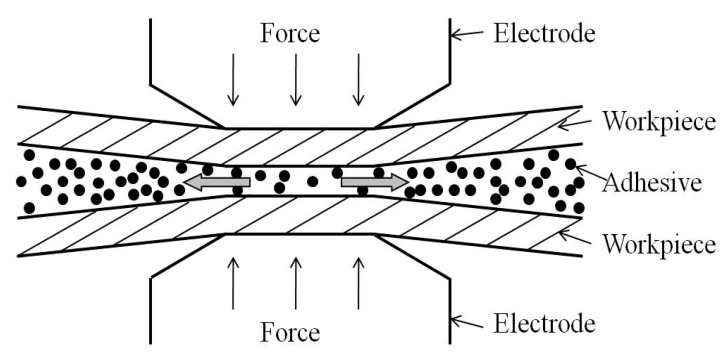
Diagram of adhesive flowing between steel sheets in the squeeze cycle.

**Figure 8:**
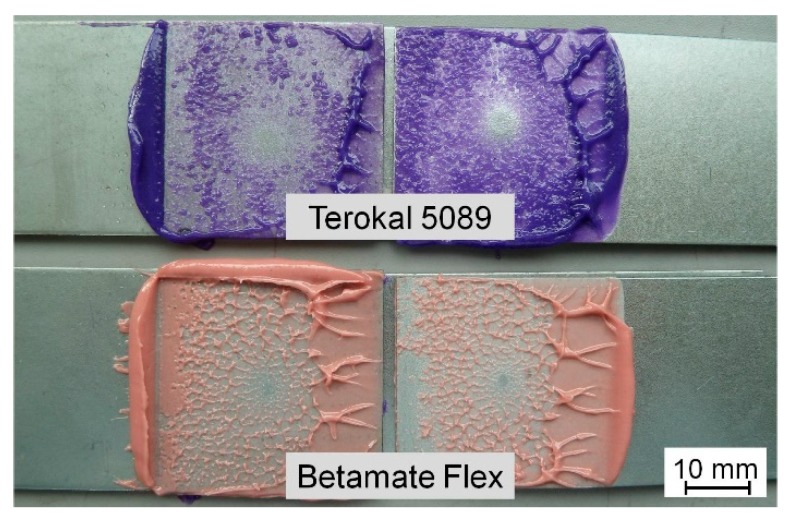
Distribution of adhesive Betamate Flex and Terokal 5089 after being squeezed.

The adhesive would flow and the bond line thickness would decrease when the electrode force is applied on the steel sheets in the squeeze cycle. At the same time, the steel sheets would deform a little and, consequently, the distribution of the adhesive would become uneven during the squeeze cycle. Figure 7 shows the general flow pattern of the adhesive and the deformation of the steel sheet under the effect of the electrode force. As shown in Figure 7, the adhesive would flow out of the faying area until two work pieces contacted directly and tightly. However, due to the viscosity of the adhesive, it could not be squeezed clearly out of the contact area. Figure 8 shows the flowing distribution of the Terokal 5089 and the Betamate Flex adhesive on the SAE1004 steel after being squeezed by an electrode force of 5.5 kN. It could be seen that the adhesive distributed mainly on the surface between the faying areas.

**Figure 9:**
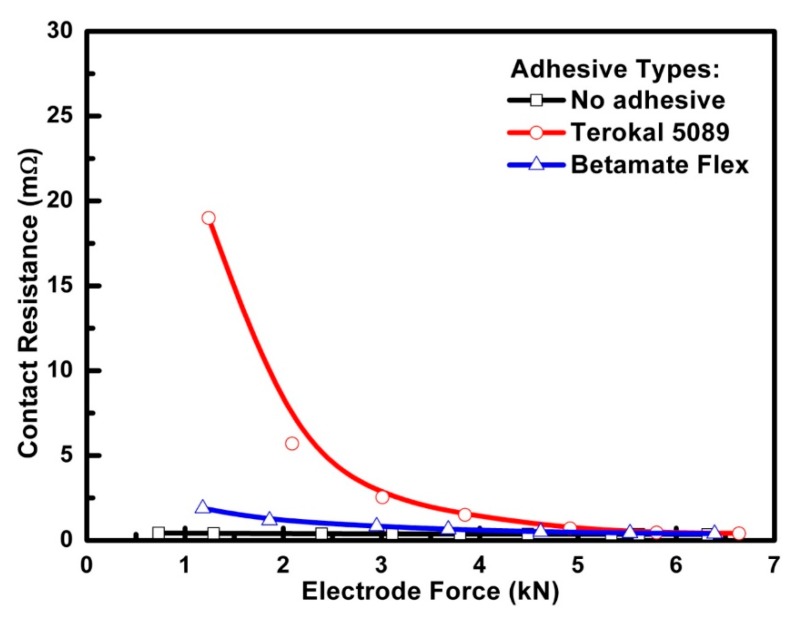
Effect of the adhesive types on the contact resistance between the faying interfaces.

In order to analyze the effect of the adhesive on the static contact resistance between the steel sheets, a test system for a contact resistance measurement was employed and the detailed introduction of test procedures is seen in our previous published paper [13]. In this part, a test result using two 0.8-mm-thick SAE1004 steels and Terokal 5089 adhesive squeezed by a different electrode force was shown in Figure 9. It could be seen that the static contact resistance between the steel materials without an adhesive is very stable when compared with Terokal 5089 when the electrode force varied from 0 kN to 6.5 kN. However, the static contact resistance with an adhesive clearly decreased when the electrode force increased. When the electrode force was greater, the static contact resistance at the faying interface will become more stable regardless of the existence of an adhesive. Figure 9 also showed that these two contact resistances would be nearly the same when the electrode force reached 5.5 kN. This test result agreed well with the analysis based on Equation (2).

**Figure 10:**
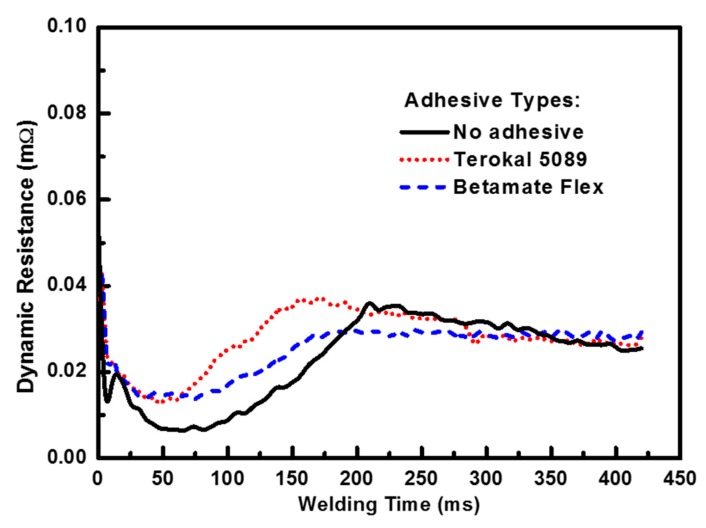
Effect of the adhesive types on the dynamic resistance during the welding process.

The change of the dynamic resistance in the weld-bonding process shows the variation of the weld nugget formation indirectly. The detailed measurement procedure of the dynamic resistance is presented by our previous published paper [13]. The dynamic resistances were prepared by the average value of five replicates for each test. The test result using two 0.8 mm thick SAE1004 steel and Terokal 5089 adhesive with a welding current of 8.0 kA was shown in Figure 10. It could be seen that the dynamic resistance curve during the first and second stage in weld-bonding was located upon the curve without an adhesive while the two curves during the third stage were similar. The great dynamic resistance in the weld-bonding process comparing to no adhesive was caused by the effect of the adhesive on the contact resistance between the faying interfaces. However, when the temperature of the work pieces went up, this effect would be reduced due to the thermal degradation of the adhesive. On the other hand, the heat generation would result in the increase of the bulk resistivity of the work pieces. Therefore, the total dynamic resistance of the faying interfaces would also be greater than without the adhesive. This result agreed well with the theoretical analysis provided by Equations (3) and (4). The dynamic resistance curves in Figure 10 also showed that the key difference between the welding process with and without an adhesive was the initial time when the weld nugget began to form.

### 5.2. Nugget Formation

**Figure 11:**
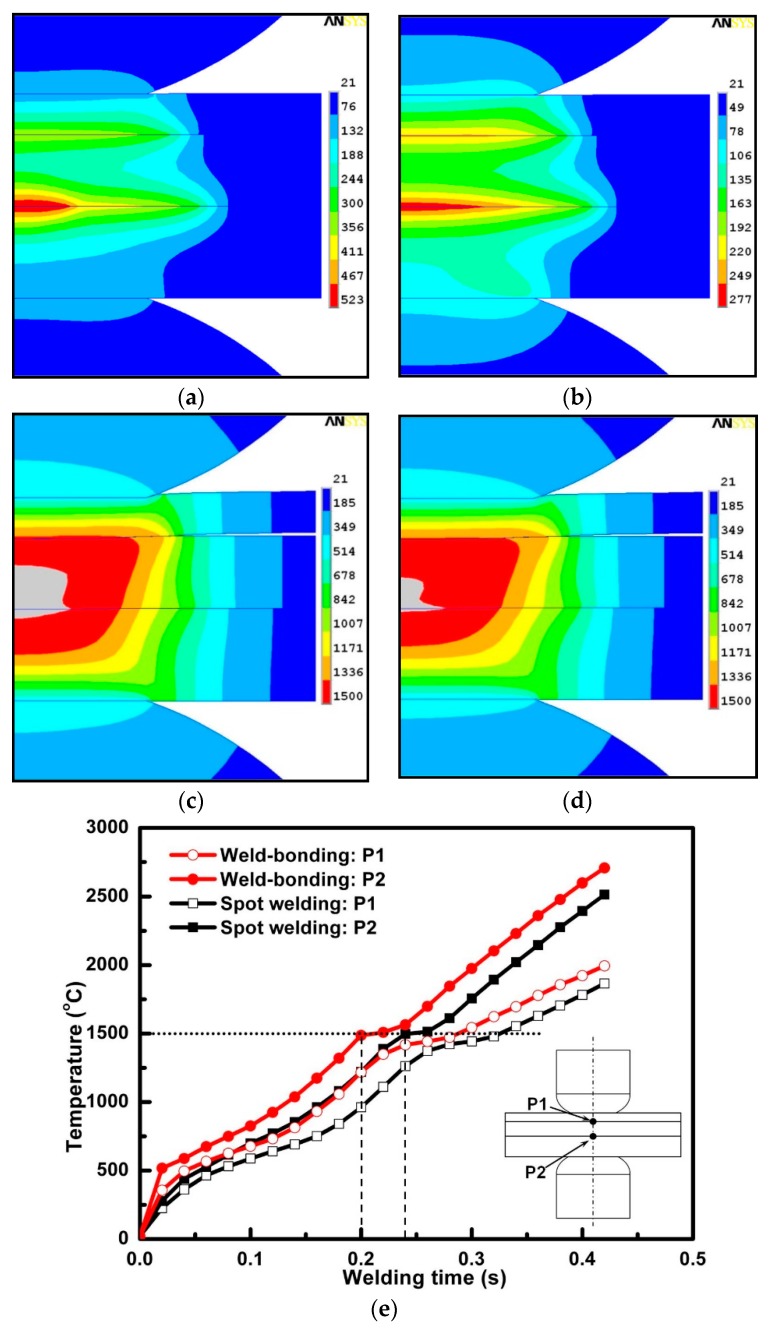
Comparison of the calculated temperature histories between the weld-bonding of Terokal 5089 and the spot welding (Legends in a–d donates the temperature with degree °C). (**a**) weld-bonding @ 20 ms; (**b**) spot welding @ 20 ms; (**c**) weld-bonding @ 240 ms; (**d**) spot welding @ 240 ms; (**e**) Calculated temperature histories of weld-bonding and spot welding.

During the welding stage, the materials properties of the work pieces and adhesive will change with the elevated temperature resulting from the heat generation. When the temperature increases to 1500 °C, a molten weld nugget would form. Figure 11 showed the comparison of the temperature histories and distributions of the weld-bonding and spot welding during the weld cycle. Figure 11a,b were the initial temperature distributions in the joint when the welding time is 20 ms. It could be seen that the temperature was centralized around the two faying interfaces in both weld-bonding and spot welding. However, the heat in the weld-bonding process was more concentrated than that in spot welding. The maximum temperature at the center point of the faying interfaces was higher in weld-bonding. This phenomenon was always kept until the temperature reached the melting point of the steel (about 1500 °C). The calculated result in Figure 11c and Figure 11d showed that, when the welding time is 240 ms, the nugget in both weld-bonding and spot welding had begun to form around the faying interface between the middle and bottom sheets. The difference between them is that the size of the weld nugget in weld-bonding was bigger than that in the spot welding. It is caused by the earlier arrival of the steel temperature to the melting point in weld-bonding in which the joule heat would generate more as a result of the great dynamic resistance compared to spot welding. The temperature history curves in Figure 11e showed that the initial time of the weld nugget was about 40 ms earlier in weld-bonding than that in RSW. This would give the weld nugget more time to grow up and consequently get bigger weld sizes in the weld-bonding process.

**Figure 12:**
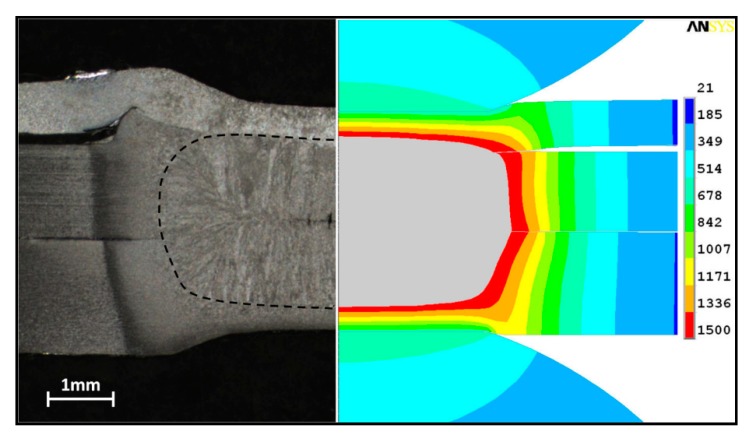
Comparison of modeling and experimental results in the weld-bonding process.

The finite element model was employed to analyze the temperature distribution during the weld-bonding process. Figure 12 shows the comparison of calculated and measured nugget formation with a welding time of 420 ms. As shown in the figure, when the calculated temperature exceeded the melting point of 1500 °C of the steel material, the zone in the finite element modeling was colored in gray, which indicated the area of the melting region. The calculated size and shape of the molten region showed good agreement with the cross-sections of the weld-bonded joint. The repetitive tests for the cross-section of the weld-bonded joint was used to verify the finite element model. As shown in Table 3, the error between modeling and the experiment is within the 5% that could be acceptable for the predicted accuracy. The weld size predicted by the model slightly underestimated the measured weld size. A possible explanation for the underestimation might lie in low contact resistance caused by the simplification of the adhesive properties used in the calculations.

**Figure 13:**
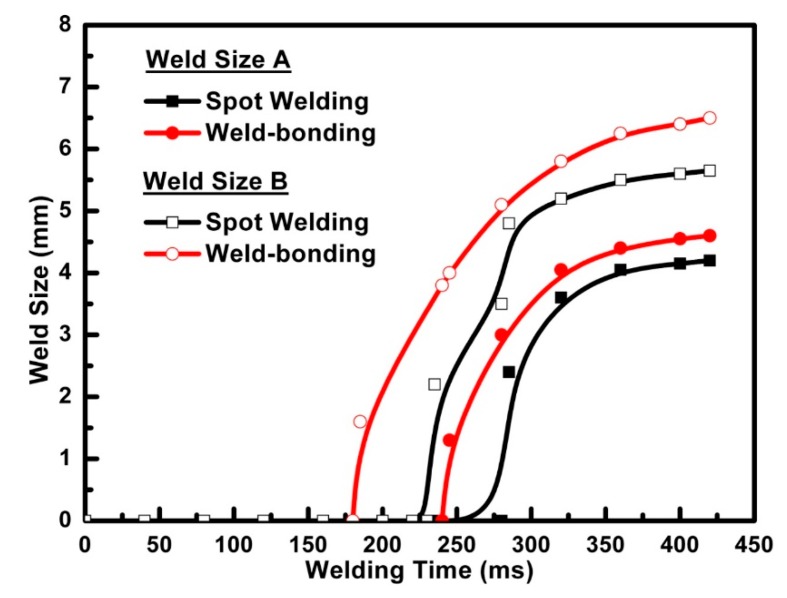
Comparison of the weld nugget formation between the weld-bonding and the spot welding.

To further analyze the nugget formation in weld-bonding of three stacks of sheets, the two weld nugget sizes of weld-bonding and spot welding with different welding time were measured by the cross-section test shown in Figure 13. The results showed that the nugget sizes A and B in the weld-bonding process began to form at about 160 ms and 240 ms, respectively. However, the initial time of the weld nugget sizes in RSW was about 220 ms and 270 ms, respectively. Consequently, the final weld nugget sizes in welding time with a 420 ms were bigger than that of spot welding due to the early formation and long time to raise the weld nugget. This proved that the adhesive would greatly influence the weld nugget formation in the weld-bonding because of the change of the heat generation around the faying interfaces and the final weld sizes in weld-bonding would increase when compared to that in spot welding.

### 5.3. Effect of Adhesive Types and Thickness on Nugget Size

**Figure 14:**
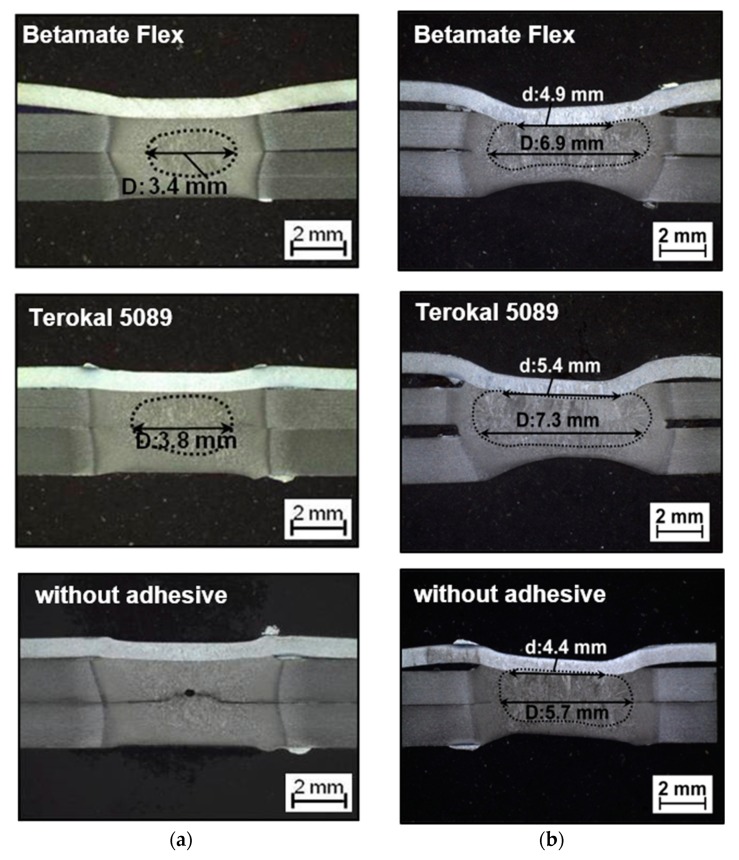
The cross-sections of the weld size in different times for different adhesive types. (**a**) 220 ms; (**b**) 420 ms.

Since the welding process of multiple stacks of steel sheets would change greatly by the placement of the adhesive [13], it was essential to study the influence of different types of adhesives on the nugget formation. Figure 14 showed the comparison during the different weld-bonding stages of a weld-bonding joint in Figure 3 with Terokal 5089 and Betamate Flex. The metallographic pictures in Figure 14 validated the different weld nugget formations with Terokal 5089 and Betamate Flex. The results showed that the weld nugget in weld-bonding with Betamate Flex initiated later than that with Terokal 5089 and consequently the final weld sizes with Betamate Flex was smaller. However, the weld nugget sizes with either Betamate Flex or Terokal 5089 were bigger than without the adhesive.

**Figure 15:**
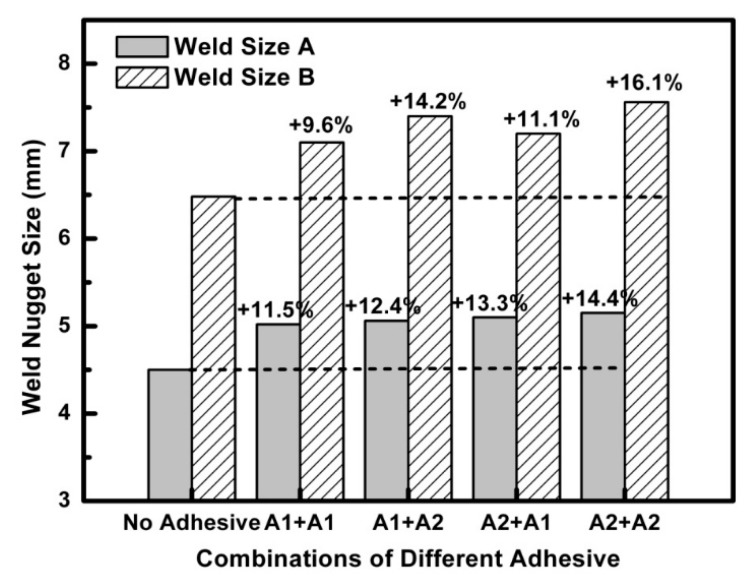
Weld nugget sizes of weld-bonding with Betamate Flex and Terokal 5089.

To investigate the effect of different types of adhesives on the weld sizes in the weld-bonding process, four different adhesive combinations with Terokal 5089 and Betamate Flex in a weld-bonding joint with three steel sheets are listed in Table 4. Five specimens were made for each kind of adhesive combination under the same welding process parameters. The results are shown in Figure 15. It could be seen that the weld nugget sizes of four combinations with adhesives were obviously bigger than without the adhesives. The average increment of the weld nugget size A and B with Terokal 5089 and Betamate Flex was 11.5–14.4% and 9.6–16.1%, respectively. Comparing the adhesive combinations “A1 + A1” and “A2 + A2,” the weld-bonding joint with Terokal 5089 had a greater increment of weld nugget sizes than that with Betamate Flex. This is due to contact resistance between the steel sheets with Terokal 5089 would have greater increment than that with Betamate Flex. Therefore, the weld-bonding joint with Terokal 5089 would generate more joule heat during the welding stage to help the weld nugget form and grow up.

**Figure 16:**
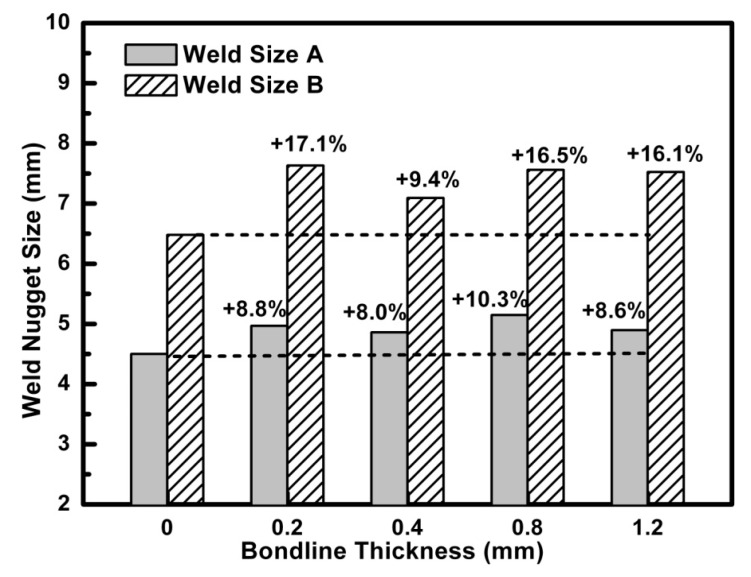
Weld nugget sizes of weld-bonding with different bond line thicknesses of Terokal 5089.

Besides the influence of the adhesive types, bond line thickness of the adhesive in the weld-bond joint is another important processing factor. Figure 16 showed the experimental results about the weld nugget sizes of weld-bonding with bond line thickness from 0.2–1.2 mm of Terokal 5089 with the same welding parameters. It could be seen that the weld sizes A with different bond line thicknesses of Terokal 5089 were dissimilar and had about a 15% increment when compared to that without an adhesive. Similar results can be found about the weld nugget size B. These results also proved that the bond line thickness of the adhesive had little effect on the weld sizes in the weld-bonding process.

## 6. Conclusions

(1) The contact resistance between the faying interfaces in weld-bonding are higher than that in resistance welding due to the viscosity of the adhesive. The critical electrode force of 5.5 kN can make the adhesive distributed mainly on the surface between the faying areas.

(2) The temperature history curves showed that the initial time of the weld nugget was about 40 ms earlier in weld-bonding than that in resistance spot welding under the same welding parameters. This would give the weld nugget more time to grow up and consequently get bigger weld sizes in the weld-bonding process.

(3) The final weld sizes in weld-bonding of three sheets are bigger than that in resistance welding. The weld-bonded joint with Terokal 5089 and Betamate Flex have about 15% and 10% increments of weld sizes, respectively. The bond line thickness of the adhesive had little effect on the weld sizes in the weld-bonding process.

## Figures and Tables

**Table 1:** Chemical composition in wt % and mechanical properties of the steel materials.

Steel	Chemical Composition in wt %						Mechanical Properties		
	C	Mn	P	S	Si	Al	Yield Strength (MPa)	Tensile Strength (MPa)	Elongation (%)
SAE1004	0.037	0.21	0.01	0.02	0.018	0.04	152	278	66
DP600	0.08	1.74	0.012	0.003	0.016	0.041	316	607	29
DP780	0.15	1.80	0.004	0.016	0.010	0.048	508	834	26

**Table 2:** Material properties of two adhesives used in this study.

Adhesive	Specific Gravity	Viscosity @50 °C (Pa·s)
Terokal 5089	1.05–1.20	30–50
Betamate Flex	1.03	20–40

**Table 3:** Comparison of the weld sizes between the modeling and experimental results.

Weld Size A (mm)			Weld Size B (mm)		
Experimental	Modeling	Error (%)	Experimental	Modeling	Error (%)
5.02	4.88	2.79	6.45	6.25	3.10
5.10		4.31	6.56		4.73
4.95		1.41	6.38		2.04
5.03		2.98	6.39		2.19
4.96		1.61	6.51		3.99

**Table 4:** Adhesive combinations with Betamate Flex and Terokal 5089.

Adhesive Combinations	Interface Between Top and Middle Sheets	Interface Between Middle and Bottom Sheets
Without adhesive	Without adhesive	Without adhesive
A1 + A1	Betamate Flex	Betamate Flex
A1 + A2	Betamate Flex	Terokal 5089
A2 + A1	Terokal 5089	Betamate Flex
A2 + A2	Terokal 5089	Terokal 5089
